# A rare case of malignant granular cell tumor of the cheek in a 16-year-old child: a case report

**DOI:** 10.1097/MS9.0000000000001077

**Published:** 2023-07-24

**Authors:** Mohanad Saleh, Husein Ahmad Sarahneh, Sara Ibrahim Hroub, Laila khader Diab, Taha mohammad Elatawneh, Saida Basem Wredat, Amal Nasr Abubaker

**Affiliations:** a Dura Hospital; b College of Medicine and Health Sciences, Palestine Polytechnic University, Hebron, Palestine

**Keywords:** case report, cheek, malignant granular cell tumor, malignant skin lesions, young

## Abstract

**Introduction and importance:**

Granular cell tumors (GCTs) are uncommon soft tissue tumors, predominantly benign lesions. Approximately 50% occur in the tongue, with a peak incidence in the fifth and sixth decades of life. However, in this case, a rare presentation of GCTs on the cheek of a 16-year-old young female, including a review of the literature.

**Methods:**

The medical records and histopathological slides of the case were retrospectively reviewed. This work has been reported based on Surgical CAse REport (SCARE) criteria.

**Case presentation:**

A 16-year-old female presented with a non-painful exophytic and pigmented cheek lesion that is rapidly growing. A primary concern was expressed as cosmetic in nature, a biopsy of the mass was taken, and histopathological findings showed a malignant tumor, mostly consistent with GCTs. She was recommended to have a total excision of the mass. During follow-up, facial MRI findings indicate granulation tissue versus recurrent/residual tumor at the site of surgery, without any evidence of cancer metastasis or spread.

**Clinical discussion:**

GCT is a tumor of Schwann cells, which is mostly benign with a 2% risk of malignant transformation. The peak incidence of this tumor is around the age of 50, and it is uncommon in young individuals. Clinically, GCT presents as a single, asymptomatic dermal or subcutaneous, brown-red nodule or papule, which grows slowly with diameter ranging from 0.5 to 3 cm. The definitive treatment for both benign and malignant GCTs is sufficient local excision with safe margins.

**Conclusion:**

GCT is an extremely rare tumor, with a peak incidence of fifth and sixth decay of age, which usually appear in the tongue (50% of cases); however, in this case, we present a 16-year-old female with a cheek mass diagnosed as GCT. In short, we think that GCT should be considered as one of the differential diagnoses of solitary facial masses at young ages.

## Introduction

Highlights

- Presentation of primary granular cell tumor (GCT) in young patients is extremely rare.
- GCT mostly comes as the mass tongue (50% of cases).
- GCT could be considered in the differential diagnosis of a solitary lesion on the cheek of a young patient.

Granular cell tumors (GCTs) are relatively uncommon soft tissue tumors, predominantly benign lesions. Most GCTs occur in the head and neck region, with ~50% of cases affecting the tongue. Other sites of occurrence include the oral cavity, larynx, bronchus, gastrointestinal tract, and breast. GCT can affect both sexes and individuals of any age, although it is most commonly found in females and dark-skinned populations. The majority of GCTs occur during the fourth to sixth decades of life, with congenital cases being extremely rare. Malignant GCTs are exceptionally uncommon, accounting for only 1–2% of cases, while multiple GCTs are observed in up to 10% of cases. In children, there have been only 20 reported cases in the literature. The gold standard for diagnosing GCT is histopathology. This report presents a rare case of a 16-year-old female who presented to the hospital with a rapidly growing, painless, red mass on her left cheek, which was diagnosed as a malignant GCT. The paper discusses the clinical presentation, histopathological findings, and the patient’s clinical progress following the surgical removal of the tumor^1,2,4–6^.

## Patient information

The patient’s main complaint is cosmetic in nature. Her medical and surgical history is unremarkable, and have no known drug allergies or significant medication use. She is a non-smoker with a good socioeconomic status. Family history is negative for malformations or genetic abnormalities.

## Case presentation

A 16-year-old female with a free past medical and surgical history has complained of a red, painless mass on her left cheek since May 2021. She described that the mass would appear and disappear intermittently over the past year. However, she noticed that recently the mass had grown rapidly and became more prominent, accompanied by increased skin discoloration compared to previous episodes. The patient’s sole concern was cosmetic.

During the examination, a pink, shiny mass with a yellowish area in the central part was observed. The patient was referred for an excisional biopsy of the lesion with a 0.1 cm margin. The resulting defect was closed primarily, and the excised tissue was sent for histopathological evaluation. The macroscopic description indicated a non-oriented ellipse of skin and underlying soft tissue, measuring 2×1.5×1.5 cm. Upon sectioning, a tan, firm lesion measuring 2×1.4×1.2 cm was identified in the subcutaneous tissue, pushing up against the epidermis.

Microscopic examination has revealed dermal-based neoplasm, composed of nests of monomorphic population of ovoid cells with round to oval nuclei, open nuclear chromatin, prominent eosinophilic nucleoli and moderate amount eosinophilic cytoplasm with little fibrocollagenous stroma, high mitotic activity, including atypical forms (>12/HPF) and foci of tumor necrosis. Lympho-vascular invasion is also noted. The tumor cells were positive for SOX10, S100, and CD68 immunostains and negative for MART1, Melan-A, HMB45 (excluding melanocytic lesion). Myoepithelial markers including P63, CK5/6 and GFAP were also negative (excluding myoepithelial carcinoma). Pan-CK, Actin, TFE3, Androgen receptor, and CD10 were also negative. Molecular and cytogenetic studies for ESWR1 gene rearrangement is performed and was negative (excluded the possibility of melanoma of soft part) (clear cell sarcoma).

**Figure 1:**
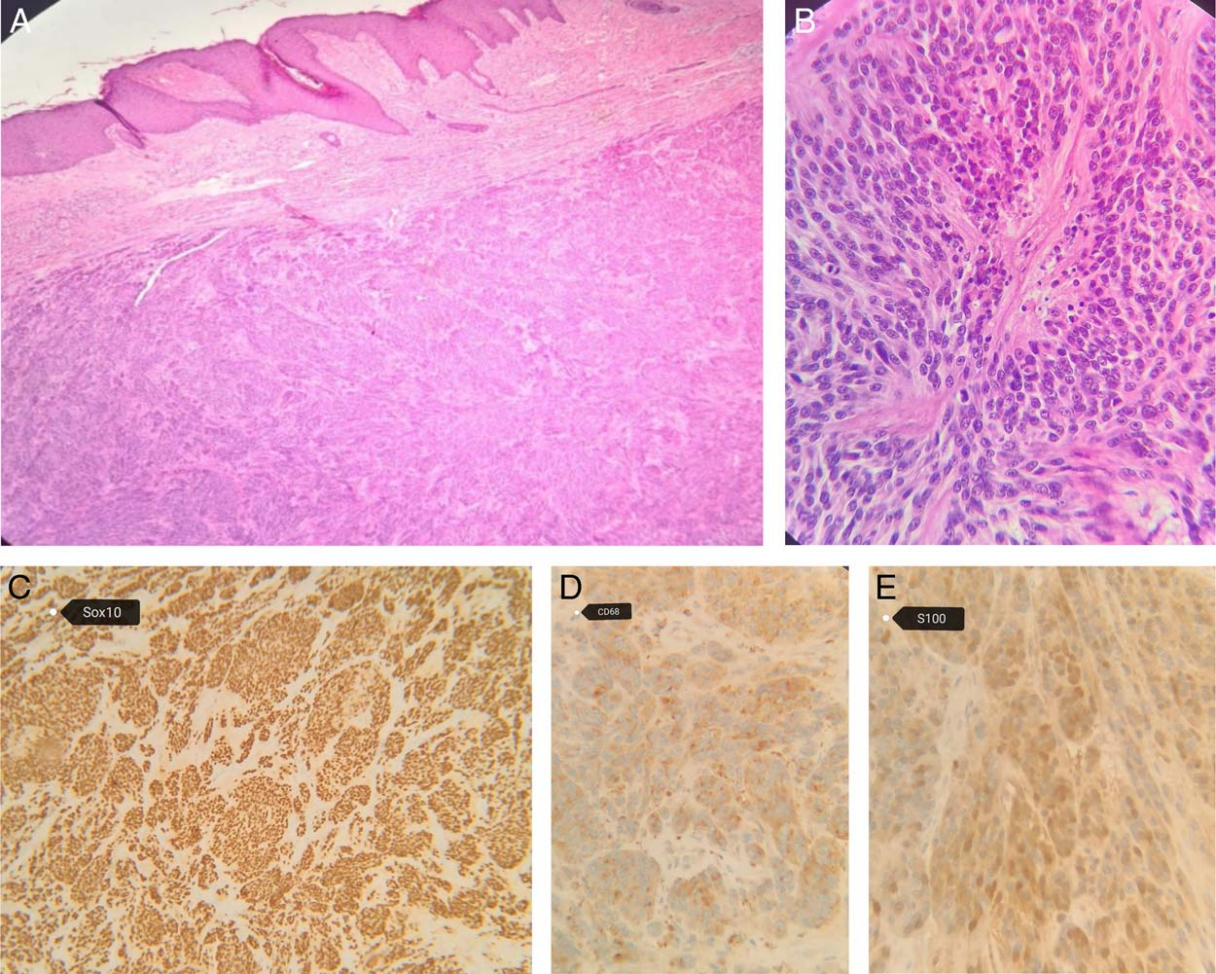
(A) Hematoxylin and eosin (H&E) stain, low-power view: dermal-based tumor with no epidermal connection. (B) H&E stain, high-power view: the tumor is composed of nests of plasmacytoid cells with ovoid nuclei, open nuclear chromatin, and prominent eosinophilic nucleoli with eosinophilic cytoplasm. (C) SOX10 immunostain is positive. (D) CD68 immunostain is positive. (E) S100 immunostain is positive.

See Figure 1 which shows H&E (hematoxylin and eosin) and S0X10, CD68 and S100, respectively.

## Intervention

The mass was successfully excised with a safety margin by a plastic surgeon and their team at a local hospital. The patient underwent the surgery without any complications and remained stable with normal vital signs. There were no signs of fever or vomiting, and her pain was effectively controlled. The patient was able to ambulate, void, and tolerate her diet. She was discharged home and will be followed up at the outpatient clinic.

## Post-intervention considerations and follow-up

Panadol (500 mg two tablets/6 h PO) and Fucidine ointment (Q1/12 h locally).

The patient had a pet-CT scan and MRI, and findings indicate no evidence of metastatic disease or cancer spread.

The patient has been advised to undergo a follow-up examination at 6-month intervals to monitor for the presence of recurrence, wound care, and medication usage. This proactive approach aims to ensure early detection and appropriate management of any potential complication related to surgery or recurrence of the lesion. The patient demonstrated high levels of intervention adherence by following the postoperative instructions and exhibited excellent tolerability, as evidenced by the absence of significant complications or any adverse effects during the recovery period.

As of June 2023, the patient’s condition remains favorable, exhibiting no indications of recurrence. The absence of any signs of recurrence in the patient’s current state suggests a positive outcome following the treatment.

## Discussion

GCT, also known as granular cell myoblastoma, is a rare tumor that arises from Schwann cells. This tumor was first described by Abrikosoff in 1926^3–9^. The majority of cases present as benign tumors, with less than 2% being malignant^2,4,5,8^. GCT is rare in children while it is more common in adults aged 30–60, with a peak of incidence at the age of 50, and more common in women and people of African-American ethnicity^5,6,8^. About 40–60% of GCT cases occur in the head and neck, with approximately half of them in the tongue^4,6,8,9^. It is also possible to occur in the breast, GI tract, respiratory tract, extremities, pituitary and parotid glands, skeletal muscles, and eyes^4,6,8,9^.

Clinically, GCT presents as a single, asymptomatic dermal or subcutaneous, brown-red nodule or papule, which grows slowly with a diameter range from 0.5 to 3 cm^3,4,6,8^. The presentation of multiple lesions of GCTs is rarely reported, and when it occurs, it is associated with neurofibromatosis and Noonan syndrome, cardiovascular and musculoskeletal abnormalities, especially in children^3,4,7^.

Histologically, the boundaries of the cells are usually indistinct and appear syncytial. The tumor cells have abundant granular eosinophilic cytoplasm with small, vesicular, or pyknotic nuclei, which may be centrally or eccentrically located^3,6–8^. The cells are arranged in sheets or fascicles with variable stroma^3,7^. The granular appearance of the cytoplasm is due to the presence of large lysosomes that stain positively with periodic acid-Schiff stain^3,6^.

There are three possible histological results of the biopsy: benign, atypical, and malignant. Six histological criteria are used to distinguish between them, including spindling, vesicular nuclei with large nucleoli, necrosis, increased mitotic activity (>2 mitoses in 10 high-power fields at 200× magnification), pleomorphism, and high nuclear to cytoplasmic ratio. If three or more of these criteria are met, the diagnosis is malignant; if one or two are met, the diagnosis is atypical; and if none of the criteria are met, the diagnosis is a benign GCT. However, these histological criteria alone are not sufficient to distinguish between them^6,9^.

In immunochemistry, typically, both benign and malignant GCTs are positive for S100, CD68, CD57, inhibin, neuron-specific, calretinin, CD56, TFE3, SOX10, PGP9.5, and vimentin^10^. However, they are negative for GFAP, CK, NF, EMA, HMB45, chromogranin, synaptophysin, and desmin^5^. There is an association between the expression of p53 and the Ki-67 proliferation index with the aggressiveness of tumor growth in some cases. Positive p53 and high Ki-67 proliferation index are shown with malignant GCTs, while negative p53 and low Ki-67 proliferation index are shown with benign GCTs^6^.

The diagnosis of GCTs is based on histopathology and immunochemistry. There are many differential diagnoses of this tumor that include myofibroma, dermatofibroma, xanthogranuloma, adnexal tumor, and melanocytic nevus^7,8^.

The definitive treatment for both benign and malignant GCTs is sufficient local excision, which can also serve as a diagnostic procedure. Surgical excision with a safe and clean margin is recommended when there is no surrounding capsule^4^. If thelesion is malignant, the sentinel lymph node biopsy is recommended^4^. In case of palpable lymph node or biopsy-proven metastatic disease, lymph node dissection is recommended; rare cases of benign GCTs are reported with local recurrence, which is noted to associate with incomplete resection^9^. However, in the malignant GCTs, the local recurrence rate is up to 41% after surgical excision, and up to 62% of them develop distant metastasis, so long-term follow-up is necessary^9^.

According to existing literature, GCTs typically are not responsive to radiotherapy and chemotherapy^5^. The primary treatment modality for these tumors is surgical removal. Due to their potential for infiltration into surrounding tissues, intervention is crucial to effectively address the tumor and prevent further complications.

Complete excision with a free margin of 1 cm is regarded as the sole curative treatment for benign GCTs with the aim of preventing recurrence^5,6^. The recurrence rate varies between 2 and 8%, with histological control of the safety margin playing a crucial role; in cases where the excisional margin is affected, the recurrence rate can increase up to 20%^6^.

Overall, the prognosis for most cases of benign GCT is considered excellent.

**Figure 2:**
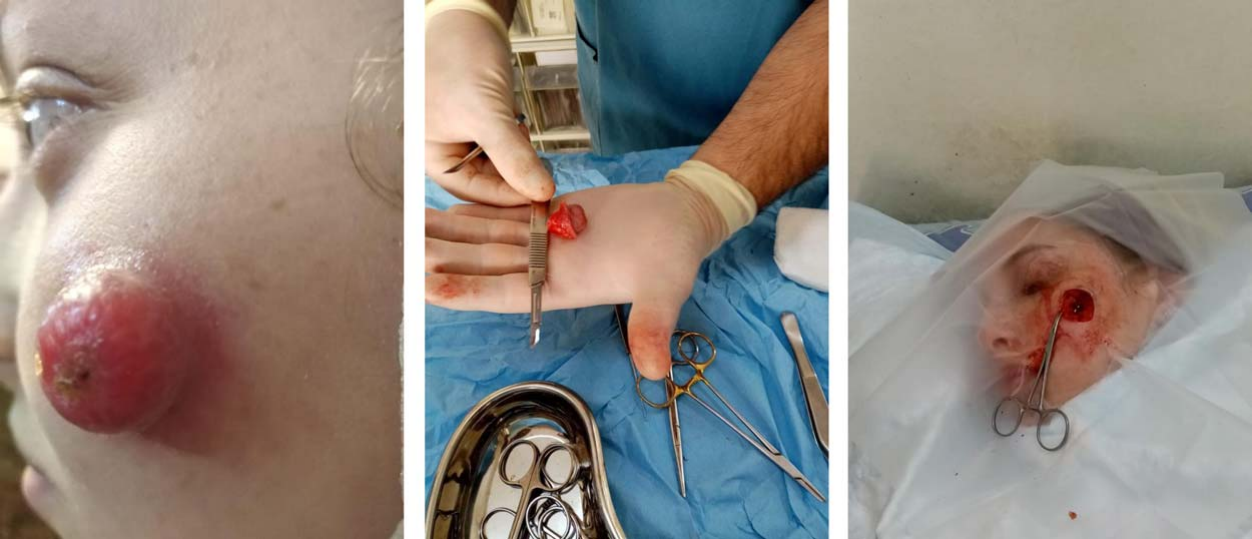
The mass before and after excision.

For this case, a total excision was performed with a free margin of 1 cm; see Figure 2.

During the follow-up, the facial MRI findings indicated granulation tissue versus recurrent or residual tumor at the site of surgery. There is focal skin thickening seen in the left cheek with underlying irregular subcutaneous fat stranding at the site of surgery, which measures around 15 mm in the maximal craniocaudal and anteroposterior dimension and around 9 mm in depth, and no associated muscular or bone marrow infiltration.

## Patient perspectives

Before the surgery, I felt really self-conscious about the growing mass on the left side of my face. I worried about complications and whether the surgery would actually remove the mass without any negative effects on how I looked or my health. I was also afraid that the mass might be connected to something more serious.

On the day I was getting ready for the surgery, my doctor explained everything to me in detail and patiently answered all my questions. The surgical team made sure I was comfortable and took good care of me.

When the day of the surgery came, I felt a mix of excitement and nervousness. But the surgical team and the anesthesiologist were very supportive, which helped calm my fears.

After the surgery, I felt a huge sense of relief when I found out that they successfully removed the facial mass. I did have some discomfort during the recovery period, but it was manageable with the pain medication they gave me.

During my recovery, I followed all the instructions from my doctor. I kept the surgical site clean and took all the prescribed medications. As time went on, the swelling went down, and I noticed a big improvement in how my face looked. This made me feel more confident and better about myself.

In conclusion, going through the facial mass surgery was a journey of feeling self-conscious and worried at first, but with the explanations from my doctor and the support from the surgical team, I was able to go through with it. The successful removal of the mass, along with taking good care of myself during recovery, made a big difference in how my face looked and how I felt about myself. Oh, and I was really happy when the PET-CT scan showed no signs of the mass spreading anywhere else in my body.

## Conclusion

GCTs are a rare neoplasm that can occur in various anatomical locations within the human body, with a notable predilection for the tongue (found in ~50% of cases). Typically, this condition manifests in older individuals, with the highest incidence observed in the fifth to sixth decades of life. However, the presented case deviates from the norm as it involves a 16-year-old patient with a lesion located on the left cheek. This occurrence represents an exceptionally uncommon presentation in this particular age group and anatomical site.

## Ethical approval

None.

## Consent

Written informed consent was obtained from the patient for publication of this case report and accompanying images. A copy of the written consent is available for review by the Editor-in-Chief of this journal on request.

## Sources of funding

No funding or grant support.

## Author contribution

M.S.: supervision; H.A.S., L.K.D., and S.I.H.: writing original draft and writing – review and editing; T.M.E.: data curation; A.N.A.: methodology; S.B.W.: resources.

## Conflicts of interest disclosure

There are no conflicts of interest.

## Research registration unique identifying number (UIN)

None.

## Guarantor

Husein Sarahneh.

## Provenance and peer review

Not commissioned, externally peer-reviewed.

## Data availability statement

The data in the current case are available from the corresponding author on request. The corresponding author has full access to all data and takes responsibility for its integrity and accuracy.
